# A predictive model for residual lesions after LEEP surgery in CIN III patients

**DOI:** 10.3389/fmed.2023.1326833

**Published:** 2023-12-11

**Authors:** Lihui Deng, Tiejun Wang, Ye Chen, Xueli Tang, Dajun Xiang

**Affiliations:** ^1^Department of Gynecology, Xishan People's Hospital of Wuxi City, Wuxi, China; ^2^Department of Gynecological Oncology, Wuxi Maternal and Child Health Hospital, Wuxi, China

**Keywords:** LEEP, post-total hysterectomy, cervical intraepithelial neoplasia grade III, predictive model, nomogram LEEP

## Abstract

**Background and aims:**

The residual lesions after Loop Electrosurgical Excision Procedure (LEEP) contributes to poor prognosis in patients with Cervical Intraepithelial Neoplasia Grade 3 (CIN3). The aim of this study is to establish an effective clinical predictive model for residual lesions in CIN3 patients after LEEP.

**Methods:**

A retrospective analysis was performed on 436 CIN3 patients who underwent total hysterectomy within 3 months after LEEP. Based on the post-hysterectomy pathologic, the patients were divided into the no residual group and residual group. Clinical parameters were compared between the two groups, and univariate and multivariate logistic regression analyses were conducted to identify independent risk factors for residual lesions in CIN3 patients after LEEP. Using R software, a nomogram model was established and its effectiveness was evaluated using calibration plots.

**Results:**

There were 178 cases in the residual group and 258 cases in the no residual group. The two groups had no significant difference in general characteristics (*p* > 0.05). It was found that Post-LEEP follow-up HPV, Post-LEEP follow-up TCT, and the Gland involvement were independent risk factors for residual lesions in CIN3 patients after LEEP (all *p* < 0.05). The consistency index (C-index) of the nomogram model for predicting residual lesions was 0.975 (0.962–0.988).

**Conclusion:**

The Post-LEEP follow-up HPV, Post-LEEP follow-up TCT, and Gland involvement are independent risk factors related to residual tissue after LEEP surgery in CIN3 patients. The constructed nomogram can effectively predict the presence of residual tissue after LEEP surgery in CIN3 patients and has good practical value.

## 1 Introduction

Cervical cancer has now become one of the most common malignant tumors among women worldwide. Its incidence and mortality rate rank fourth, accounting for 6.5 and 7.7%, respectively (1). Cervical intraepithelial neoplasia (CIN) is a precursor lesion of cervical cancer. Interventions and treatments at this stage can effectively reduce the incidence of cervical cancer (2). Currently, the preferred treatment option for CIN is LEEP which offers advantages such as minimal invasiveness, simplicity in operation, and the ability to preserve fertility (3). However, residual lesions after LEEP surgery pose a challenging clinical problem. Recent studies have shown that the residual rate of CIN after LEEP surgery remains as high as 18.2–31.1% (4, 5). Currently, there are no effective indicators available for predicting residual lesions after LEEP surgery (6). Building an effective predictive evaluation model can offer valuable guidance for the treatment of patients with CIN after LEEP surgery (7). Therefore, it would be beneficial for the clinical treatment of CIN and improve patients’ prognosis to investigate the independent risk factors for residual CIN after LEEP surgery and establish an effective clinical predictive model providing more intuitive and personalized prediction results. For this purpose, this study utilized total hysterectomy specimens to analyze the independent risk factors for residual lesions after LEEP surgery in CIN3 patients and established an effective nomogram model. The results are reported as follows.

## 2 Materials and methods

### 2.1 Study design

**Figure 1:**
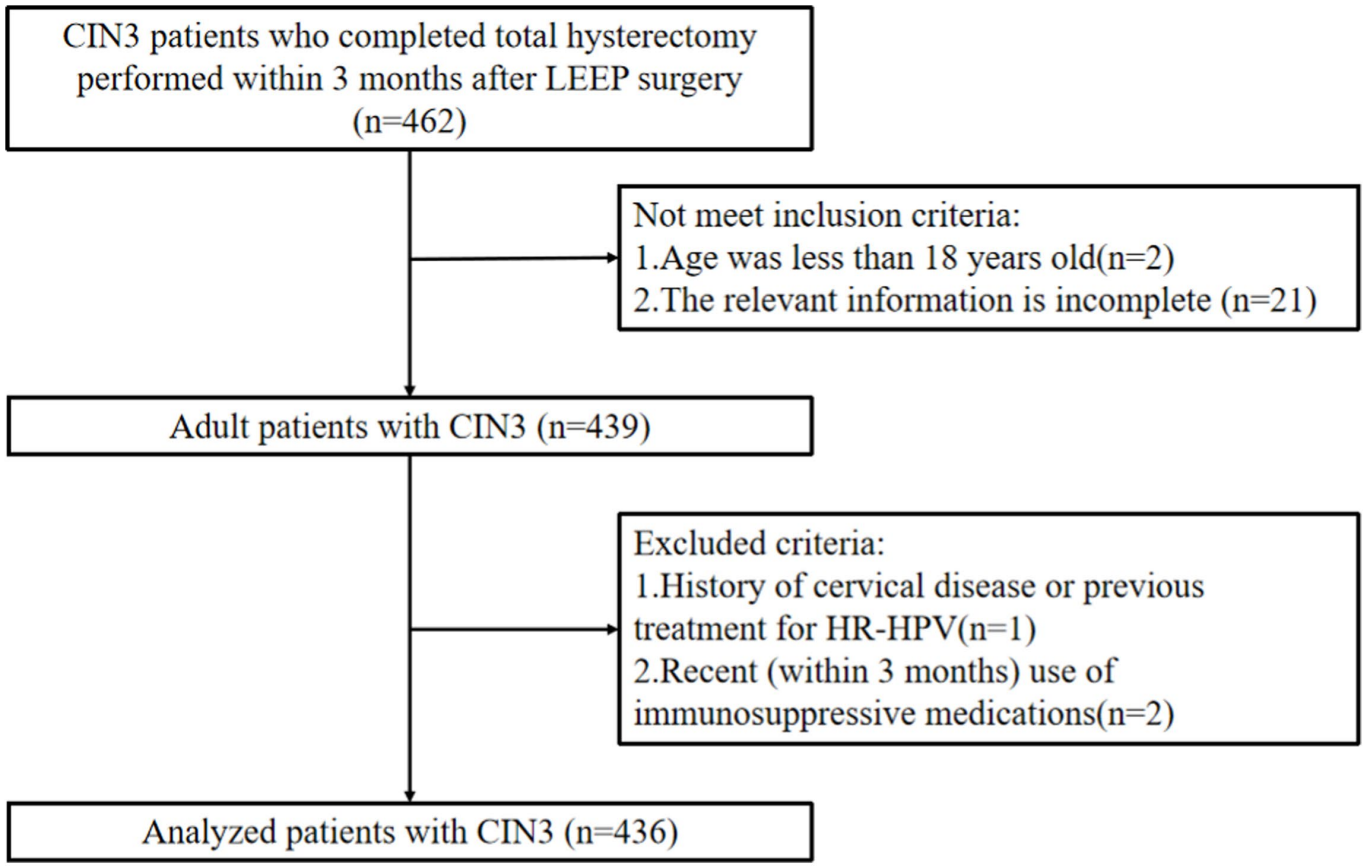
The flow chart of study design and patient selection.

The retrospective analysis was performed on 436 CIN3 patients who underwent total hysterectomy within 3 months after LEEP at Wuxi Xishan People’s Hospital and Wuxi Maternal and Child Health Hospital from January 2009 to December 2021 (see Figure 1). Inclusion criteria were as follows: (1) Patients with CIN3 who underwent LEEP surgery followed by total hysterectomy within 3 months. (2) Smooth surgical process without significant intraoperative or postoperative complications. (3) Complete relevant data available. (4) Age > 18 years. (5) Willing to participate in long-term follow-up. Exclusion criteria were as follows: (1) Pregnant or breastfeeding women. (2) History of cervical diseases or previous treatment for high-risk HPV. (3) Recent use of immunosuppressive drugs within the past 3 months. This study has obtained approval from the Independent Ethics Committee for Clinical Research of Xishan People’s Hospital (Ethics Approval Number: xs2021ky005), and informed consent has been obtained from all patients.

### 2.2 Data collection

After the patient is admitted, relevant examinations should be completed and the patient’s pre-and post-LEEP treatment hospitalization information, as well as general information, should be collected. This includes testing for HR-HPV and ThinPrep Cytology Test (TCT). TCT is divided into the following categories: A typical squamous cells of undetermined significance (ASC-US), Atypical squamous cells, cannot exclude high-grade squamous intraepithelial lesion (ASC-H), Low-grade squamous intraepithelial lesion (LSIL), High-grade squamous intraepithelial lesion (HSIL), No evidence of lesion or malignancy (NILM). For all patients who undergo hysterectomy, a pathological examination of the uterus should be performed and sent for assessment. The diagnosis of residual CIN is confirmed based on the pathological histology after hysterectomy.

### 2.3 Nomogram for individualized prediction

We used the bidirectional stepwise selection method based on Akaike’s information criteria to perform multivariable logistic regression analysis on the included CIN3 patients to identify independent risk factors for residual lesions after LEEP procedure. Variables with a *p*-value < 0.05 were defined as independent predictive factors. A random forest plot was created to display the accuracy and importance of the predictive variables, and a nomogram was constructed for individualized prediction of residual lesions after LEEP procedure. To validate the nomogram, the area under the curve (AUC) was calculated (with 1,000 bootstrap resamples) to evaluate discrimination, and calibration curve analysis (with 1,000 bootstrap resamples) was conducted to assess calibration. The calibration was statistically evaluated using the Hosmer-Lemeshow test. To assess the clinical utility of the line chart, decision curve analysis was performed to calculate the standardized net benefit at different threshold probabilities, using post-hysterectomy pathological results as the comparator for the line chart model.

### 2.4 Statistical methods

The data processing will be carried out using SPSS 20.0 statistical software. For continuous variables, they will be represented by mean ± standard deviation or median (interquartile range, IQR), depending on whether the data follows a normal distribution. To compare the means or medians between different groups, *t*-tests or Mann–Whitney U tests will be used. For categorical variables, frequencies and percentages will be provided, and Pearson’s chi-square test will be used for group comparisons. The results will be presented as odds ratios (ORs) with 95% confidence intervals (CIs). Logistic regression analysis will be used to analyze independent risk factors for Postoperative residual lesion after LEEP, considering differences with a statistical significance of *p* < 0.05. R (x64 for Windows, version 3.6.1) will be used to establish a nomogram model.

## 3 Results

### 3.1 Baseline data characteristics of the patient

This study included a total of 436 patients with CIN3 who underwent hysterectomy after LEEP. The average age of the patients was 53.6 ± 8.0 years. Among them, there were 178 cases in the group with confirmed residual lesions, with an average age of 53.8 ± 8.1 years. In the group without residual lesions after LEEP, there were 258 cases, with an average age of 53.5 ± 8.0 years. There were no statistically significant differences between the two groups in terms of age at menarche, menstrual cycle, age at marriage, parity, or pregnancy history. There were also no statistically significant differences between the two groups in terms of the presence of previous tumor history (Table 1).

**Table 1:** Baseline characteristics of patients.

	Overall (*n* = 436)	No residue (*n* = 258)	Residue (*n* = 178)	*p*-value
Age	53.6 ± 8.0	53.5 ± 8.0	53.8 ± 8.1	0.67
Age of menarche	15 (14, 16)	15 (14, 16)	15 (14, 16)	0.14
Menstrual cycle (days)	30 (30, 30)	30 (30, 30)	30 (30, 30)	0.25
Marriageable age	24 (23, 25)	24 (23, 25)	24 (23, 25)	0.36
Gravidity	3 (2, 3)	3 (2, 3)	3 (2, 4)	0.10
Parity	1 (1, 2)	1 (1, 2)	1 (1, 2)	0.83
History of tumors				0.80
NO	387 (88.8%)	231 (53.0%)	156 (35.8%)	
Benign tumor	37 (8.5%)	20 (4.6%)	17 (3.9%)	
Malignant tumor	12 (2.7%)	7 (1.6%)	5 (1.1%)	
Bleeding after intercourse				0.39
NO	397 (91.1%)	233 (53.5%)	164 (37.6%)	
Yes	39 (8.9%)	25 (5.7%)	14 (3.2%)	
Increased vaginal discharge				0.07
NO	418 (95.9%)	244 (56.0%)	174 (39.9%)	
Yes	18 (4.1%)	14 (3.2%)	4 (0.9%)	

### 3.2 Postoperative condition after LEEP

There were no statistically significant differences (*p* > 0.05) between the residual group and the non-residual group in terms of HPV status, TCT examination, LEEP pathology, and margin status prior to LEEP treatment. However, the number of cases with glandular involvement in the residual group was significantly higher compared to the non-residual group (*n* = 100 vs. 4), with a statistically significant difference (*p* < 0.001). The margin status within the LEEP specimen in the residual group also showed a statistically significant difference compared to the non-residual group (*p* = 0.001) (refer to Table 2 for more details).

**Table 2:** Post-LEEP pathological findings.

	Overall (*n* = 436)	No residue (*n* = 258)	Residue (*n* = 178)	*p*-value
HPV infection before LEEP				0.12
Negative	87 (20%)	57 (13.1%)	30 (6.9%)	
Positive	111 (25.5%)	68 (15.6%)	43 (9.9%)	
Other 12 positive	108 (24.8%)	67 (15.4%)	41 (9.4%)	
16/18 positive	130 (29.8%)	66 (15.1%)	64 (14.7%)	
TCT before LEEP				0.72
NILM	124 (28.4%)	76 (17.4%)	48 (11%)	
ASC-US	93 (21.3%)	59 (13.5%)	34 (7.8%)	
ASC-H	67 (15.4%)	36 (8.3%)	31 (7.1%)	
LSIL	47 (10.8%)	26 (6%)	21 (4.8%)	
HSIL	105 (24.1%)	61 (14%)	44 (10.1%)	
Pathology of LEEP				0.75
Normal or inflammation	57 (13.1%)	36 (8.3%)	21 (4.8%)	
LSIL	46 (10.6%)	27 (6.2%)	19 (4.4%)	
HSIL	324 (74.3%)	191 (43.8%)	133 (30.5%)	
Suspected of an early invasive carcinoma	9 (2.1%)	4 (0.9%)	5 (1.1%)	
LEEP internal cutting margin				0.001
Normal or inflammation	234 (53.7%)	152 (34.9%)	82 (18.8%)	
LSIL	38 (8.7%)	27 (6.2%)	11 (2.5%)	
HSIL	164 (37.6%)	79 (18.1%)	85 (19.5%)	
LEEP outer cut margin				0.47
Normal or inflammation	294 (67.4%)	169 (38.8%)	125 (28.7%)	
LSIL	45 (10.3%)	30 (6.9%)	15 (3.4%)	
HSIL	97 (22.2%)	59 (13.5%)	38 (8.7%)	
Gland involvement				<0.001
NO	332 (76.1%)	254 (58.3%)	78 (17.9%)	
Yes	104 (23.9%)	4 (0.9%)	100 (22.9%)	

LEEP, loop electrosurgical excision procedure; TCT, Thinprep cytology test; NILM, Negative for intraepithelial lesion or malignancy; ASC-US, Atypical squamous cells of undetermined significance; ASC-H, Atypical squamous cells cannot exclude high-grade squamous intraepithelial lesion; LSIL, Low-grade squamous intraepithelial lesion; HSIL, High-grade squamous intraepithelial lesion.

### 3.3 Independent factors associated with residual lesions after LEEP

**Figure 2:**
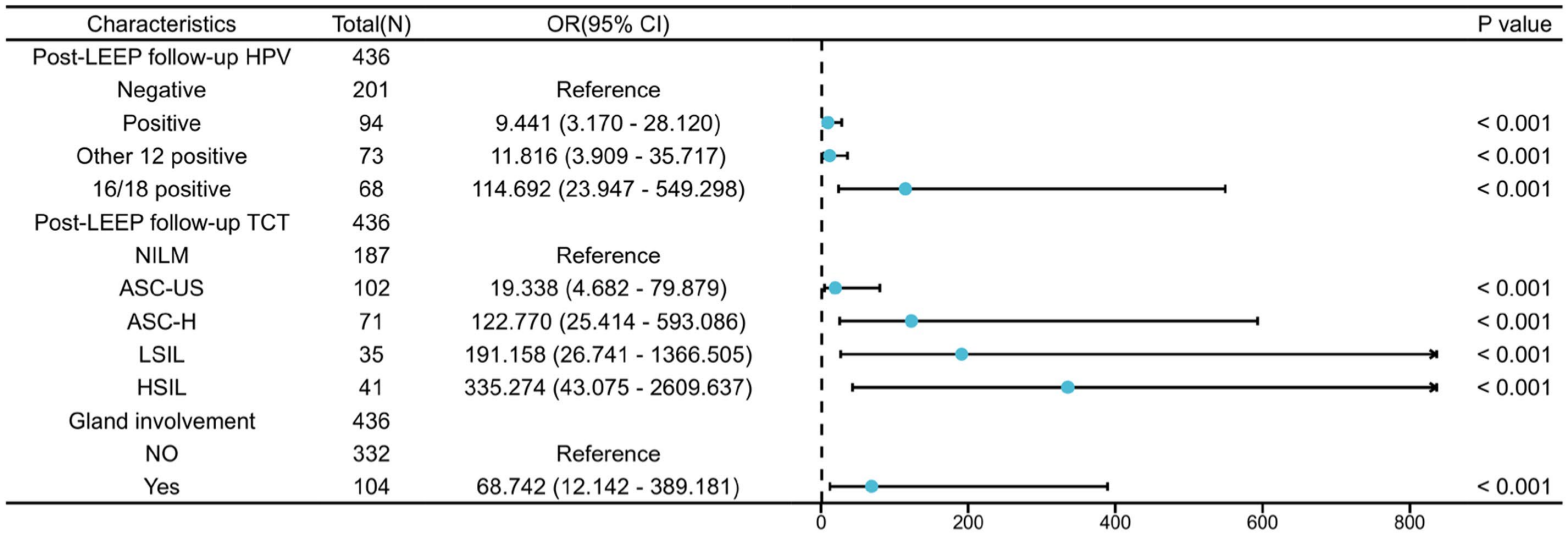
Multifactorial logistic regression analysis of residual lesions after LEEP in CIN3 patients.

Univariable and multivariable logistic regression analysis were performed on the two groups of patients (Table 3). According to the Univariable logistic regression analysis, factors such as preoperative ECC pathology, LEEP margin status, Post-LEEP follow-up HPV, Post-LEEP follow-up TCT, and the Gland involvement are potential independent risk factors for residual lesions in patients with CIN3 (all with *p* < 0.05). These variables were included in the multivariate logistic regression analysis to identify the independent factors associated with residual lesions after LEEP in CIN3 patients, including Post-LEEP follow-up HPV, Post-LEEP follow-up TCT, and the involvement of glandular lesions. To visualize the accuracy and importance of these predicting factors, a random forest plot was generated (Figure 2).

**Table 3:** Univariable and multivariable logistic regression analysis of residual factors following LEEP procedure in CIN3 patients.

Characteristics		Univariable analyses		Multivariable analyses	
	Total (*N*)	OR (95% CI)	*p* value	OR (95% CI)	*p* value
ECC before LEEP	436				
Normal or inflammation	266	Reference		Reference	
HSIL	126	4.126 (2.636–6.457)	<0.001	1.103 (0.395–3.082)	0.852
LSIL	44	2.455 (1.284–4.691)	0.007	2.016 (0.564–7.207)	0.281
LEEP internal cutting margin	436				
Normal or inflammation	234	Reference		Reference	
LSIL	38	0.755 (0.356–1.600)	0.464	1.325 (0.334–5.264)	0.689
HSIL	164	1.994 (1.327–2.997)	<0.001	2.003 (0.793–5.055)	0.141
Post-LEEP follow-up HPV	436				
Negative	201	Reference		Reference	
Positive	94	20.577 (10.396–40.729)	<0.001	9.441 (3.170–28.120)	<0.001
Other 12 positive	73	19.145 (9.359–39.166)	<0.001	11.816 (3.909–35.717)	<0.001
16/18 positive	68	213.714 (67.882–672.837)	<0.001	114.692 (23.947–549.298)	<0.001
Post-LEEP follow-up TCT	436				
NILM	187	Reference		Reference	
ASC-US	102	12.900 (6.103–27.269)	<0.001	19.338 (4.682–79.879)	<0.001
ASC-H	71	60.844 (26.109–141.788)	<0.001	122.770 (25.414–593.086)	<0.001
LSIL	35	188.800 (49.239–723.931)	<0.001	191.158 (26.741–1366.505)	<0.001
HSIL	41	224.200 (58.886–853.611)	<0.001	335.274 (43.075–2609.637)	<0.001
Gland involvement	436				
NO	332	Reference		Reference	
Yes	104	81.410 (29.042–228.210)	<0.001	68.742 (12.142–389.181)	<0.001

LEEP, loop electrosurgical excision procedure, TCT, Thinprep cytology test, NILM, Negative for intraepithelial lesion or malignancy, ASC-US, Atypical squamous cells of undetermined significance, ASC-H, Atypical squamous cells cannot exclude high-grade squamous intraepithelial lesion, LSIL, Low-grade squamous intraepithelial lesion, HSIL, High-grade squamous intraepithelial lesion.

### 3.4 Establishment and evaluation of the nomogram model

**Figure 3:**
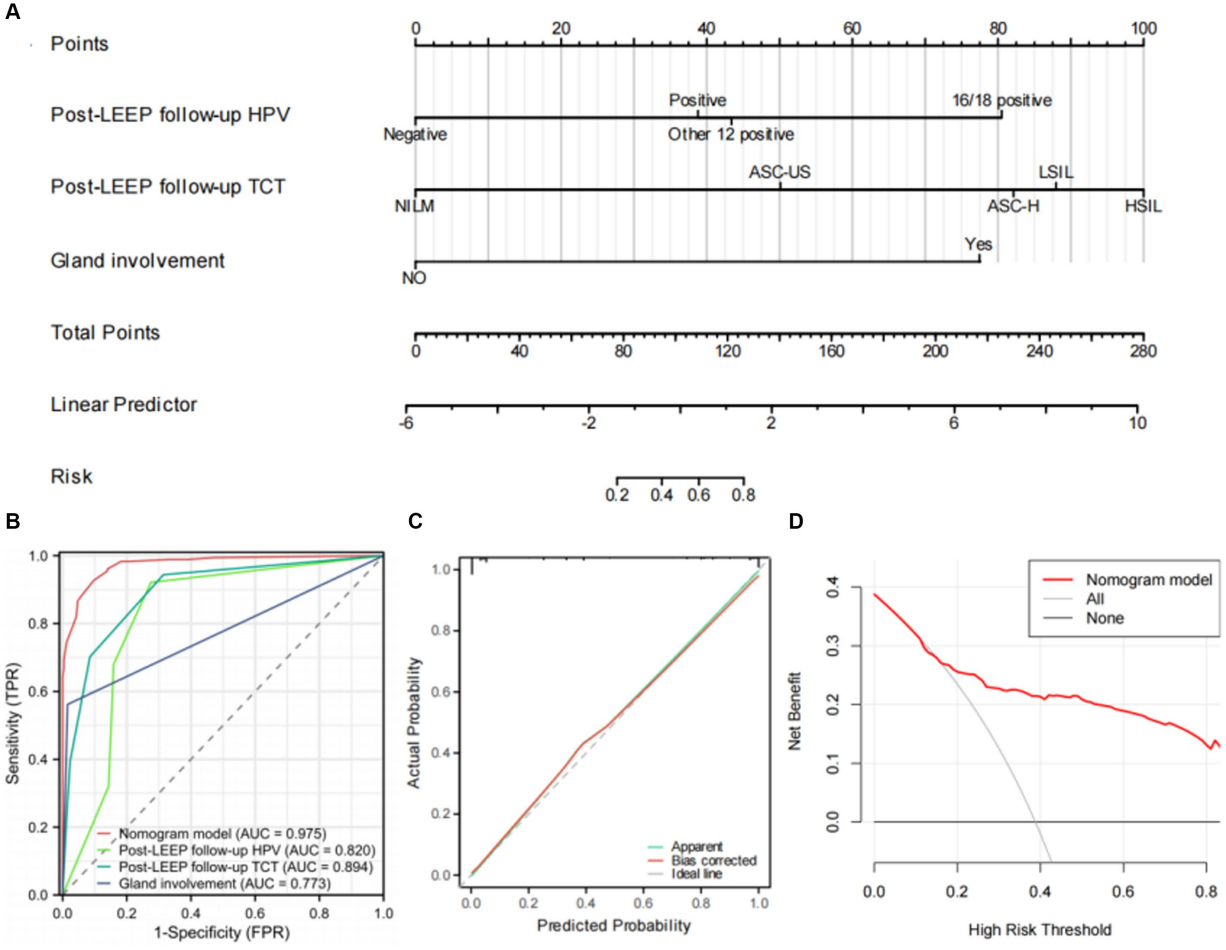
Establishment and evaluation of the nomogram. **(A)** The nomogram for predicting residual lesions after LEEP in CIN3 patients. **(B)** Area under the ROC curves (AUC) for the diagnosis of the residual lesions using the nomogram in CIN3 patients. **(C)** The calibration curve for the risk of residual lesions after LEEP surgery in CIN3 patients. The nomogram-predicted probability of residual lesions is plotted on the x-axis; the actual risk of residual lesions is plotted on the y-axis. **(D)** Decision curve analysis of the nomogram (red line).

Based on the results of the multivariable logistic regression analysis, a nomogram model for predicting residual lesions after LEEP in CIN3 patients was established using the R software (Figure 3A). The model’s discrimination was evaluated by plotting the receiver operating characteristic curve (ROC) (Figure 3B) and calculating the area under the ROC curve (AUC) and C-index. The calibration curve was also plotted to assess the consistency of the nomogram model in predicting the probability of residual lesions after LEEP. The study samples were used as the training set, and internal validation was performed using 1,000 bootstrap samples. The decision curve analysis (DCA) was conducted to evaluate the clinical benefits of the model. The results showed that the AUC values for predicting residual lesions in CIN3 patients after LEEP were 0.820 for Post-LEEP follow-up HPV, 0.894 for Post-LEEP follow-up TCT, and 0.773 for the involvement of glandular lesions. However, the nomogram model had a C-index and AUC of 0.975, indicating better predictive performance compared to individual risk factors. The calibration curve demonstrated good consistency of the model (Figure 3C). The decision curve for the training set (Figure 3D) showed that when the high-risk threshold was set to >0.18, using the nomogram model provided more benefits compared to no treatment or treating all patients. Therefore, the nomogram model has clinical value in predicting residual lesions after LEEP.

## 4 Discussion

CIN is a precancerous condition of the cervix and is one of the most common gynecological diseases among women of childbearing age. The natural course of CIN is unpredictable, and if left untreated, it can progress to invasive cervical cancer (ICC) (8). Conization of uterine cervix is a key method for diagnosing and treating CIN. The two most common techniques for conization of uterine cervix are LEEP and cold knife conization (CKC) (9). The principle of LEEP involves the use of targeted radiofrequency waves to break molecular bonds and generate heat within the tissue. This results in cutting and cauterization effects, making LEEP a minimally invasive procedure with faster recovery compared to other treatment methods (10). LEEP is highly effective for treating CIN. However, for high-grade CIN, due to its multifocal nature, complete excision through local LEEP can be challenging. This can lead to further disease progression (11). There is evidence suggesting that women who have residual lesions after treatment have a approximately 5-fold higher risk of developing invasive cervical cancer (ICC) compared to the general population (12). However, excessive excision can lead to negative consequences such as increased risk of premature births in young women, adverse outcomes for newborns, and impacts on sexual health (13, 14). Therefore, it is of great significance to establish an effective predictive model for residual lesions after LEEP to guide the treatment and prognosis of CIN patients. Currently, there is no effective evaluation system for predicting CIN residuals. The nomogram model can present the relevant factors in regression analysis in a graphical form, providing approximate probability values while integrating multiple related factors. It has good visual and operational characteristics (15).

Currently, in most studies, “positive surgical margins” and “persistent HPV infection” after LEEP are identified as risk factors for residual lesions (16, 17). Although positive surgical margins may reflect the characteristics of the disease, multiple studies have also confirmed its correlation with CIN recurrence (18, 19). However, nearly half of the cases with positive surgical margins do not experience recurrence or residual disease, and even patients with negative margins may still have residual disease (20, 21). Furthermore, in the results of this study, there was no correlation between the status of the inner and outer surgical margins after LEEP and the presence of residual lesions. Although there was a statistical difference in the status of the inner surgical margins after LEEP between the residual group and the non-residual group (see Table 2), multivariable logistic regression analysis showed that the status of the inner surgical margins after LEEP was not an independent risk factor for residual lesions (see Table 3). This study speculates whether the absence of residual lesions after hysterectomy following LEEP with positive surgical margins is related to the use of electrosurgical coagulation and hemostasis during LEEP. Other way, we do not rule out the possibility that this could be caused by collinearity between the variables, as well as potential selection bias and a relatively short follow-up period (18).

HPV infection has been confirmed by multiple studies as an important predictive indicator for residual CIN lesions (5, 22, 23). In this study, the post-LEEP follow-up HPV infection status was closely related to the presence of residual CIN3 lesions after LEEP, and it can serve as an independent predictive indicator for residual lesions in CIN3 patients undergoing LEEP. These findings are consistent with previous research. Among the 150+ known HPV subtypes, more than 40 subtypes are known to be associated with genital infections. HPV infection, especially high-risk (HR) HPV infection, is a risk factor for various gynecological diseases (24). The results of the multifactor logistic regression analysis showed that post-LEEP follow-up HPV infection, follow-up TCT results, and involvement of glandular lesions were independent risk factors for residual lesions after LEEP (*p* < 0.05, Table 3). TCT has now replaced conventional cytology as an important method for cervical cancer screening. It has high sensitivity in the diagnosis of CIN lesions (25). Based on the above results, we have established a waterfall plot model (Figure 3A) to predict the presence of residual lesions after LEEP in CIN3 patients. The nomogram model shows that using post-LEEP follow-up HPV infection, follow-up TCT results, and involvement of glandular lesions as predictive indicators has a good C-index level (0.975, 95% CI = 0.962–0.988). The calibration curve further confirmed the good correlation between the model and the actual occurrence of residual lesions in CIN3 patients post-LEEP. This indicates that the model can effectively predict the presence of residual lesions after LEEP in CIN3 patients. Additionally, clinical decision analysis shows that this model provides more benefits than having no patient treatment plan or treating all patients. It can provide valuable guidance for clinicians in preventing residual lesions after LEEP in CIN3 patients, demonstrating good clinical utility.

This study has the following limitations: the model lacks external data for evaluation and validation, which reduces the reliability and limits the generalizability of the nomogram model. Additionally, this study is a single-center retrospective study, which may result in lower generalizability of the findings. Therefore, further clinical research is needed to validate the nomogram model using multicenter, prospective studies with larger sample sizes and external data. Furthermore, the assessment of HPV-mRNA appears to serve as a prognostic biomarker for monitoring residual disease progression in women undergoing LEEP for CIN3 (26). This biomarker will be further examined and analyzed in our upcoming research endeavors.

In conclusion, the nomogram model constructed in this study based on post-LEEP follow-up HPV infection, follow-up TCT results, and Gland involvement has demonstrated some predictive performance for residual CIN3 lesions after LEEP. After further validation, it may have good clinical application prospects. Additionally, the results of this study also highlight the importance of the postoperative follow-up examination after LEEP.

## Data availability statement

The original contributions presented in the study are included in the article/supplementary material, further inquiries can be directed to the corresponding authors.

## Ethics statement

The studies involving humans were approved by IEC for Clinical Research of Xishan People’s Hospital. The studies were conducted in accordance with the local legislation and institutional requirements. The participants provided their written informed consent to participate in this study. Written informed consent was obtained from the individual(s) for the publication of any potentially identifiable images or data included in this article.

## Author contributions

LD: Data curation, Formal Analysis, Resources, Writing – original draft. TW: Data curation, Resources, Writing – original draft. YC: Data curation, Resources, Writing – original draft. XT: Conceptualization, Investigation, Methodology, Project administration, Resources, Software, Validation, Visualization, Writing – review & editing. DX: Conceptualization, Funding acquisition, Investigation, Project administration, Resources, Software, Validation, Writing – review & editing.
